# Death Instantly Following a Blow on the Mouth

**Published:** 1848-01

**Authors:** Hayes Kyd

**Affiliations:** Harefield


*Death instantly following a blow on the mouth.* By Hayes Kyd, Esq., M. R. C. S. E., L. S. A., Harefield.—In communicating the following case, I am influenced by a desire, not to impart, but to elicit information from the practical columns of The Lancet; and although the subject may not possess the claim of novelty, yet I trust it may be regarded as involving considerations of interest and importance to my professional brethren. The case is one of instantaneous death—the result of a blow received on the mouth during a pugilistic encounter. The principals were two athletic young boatmen,(James Carpenter and William Norman,) in the prime of life, and of equal age,—viz., twenty-two years. The fatal conflict occurred at Harefield Lock, on the Grand Junction Canal. Six rounds were rapidly fought in a fair, manly style, without any decisive result, both parties being fresh, and apparently but slightly injured. The contest was now adjourned, by mutual consent, to an adjoining meadow, as being a more commodious and secluded spot for deciding their differences. Here hostilities were speedily renewed, and seven more rounds had been fought. In the seventh, Carpenter struck Norman violently on the mouth. The latter instantly fell, and ere his brother could rush but a few yards to his assistance, had expired. Seventy-two hours after death, I examined the body, by the coroner's order, and observed the following appearances:—

*Sectio cadaveris.*—Body muscular and well formed ; the general appearance that of a healthy and athletic young man. Contusions of both eyes, nose, and mouth, especially about the upper lip ; marks of much effusion of blood from the nose, and considerable discharge of blood from the same cavity, on moving the body ; abrasions of the cuticle from over the right and left cheek-bones, nose, and chin, and slight abrasions on either side of the lower jaw ; pupils slightly dilated ; no external injuries perceptible on any other part of the body ; the vessels of the scalp and external pericranium congested, particularly over the occipital and temporal regions. On removing the skull, the vessels of the brain were found much congested, the sulci of the convolutions being filled up through engorgement of the pia mater ; and the lateral ventricles were found loaded with extravasated blood, from rupture of the choroid plexus. On removing the brain, the middle and posterior fossæ of the skull became instantly filled with blood, on division of the lateral and petrosal sinuses, showing extreme congestion of those venous reservoirs. No fracture of either table of the skull, nor any perceptible injury at its base ; the lungs healthy, but engorged ; heart healthy ; right side loaded with venous blood.

The opinion I formed was, that death had resulted form a violent concussion of the brain, accompanied by compression, from extravasation of blood in the brain.

